# Defining growth requirements of microgreens in space cultivation via biomass production, morpho-anatomical and nutritional traits analysis

**DOI:** 10.3389/fpls.2023.1190945

**Published:** 2023-07-19

**Authors:** Chiara Amitrano, Gabriele Paglialunga, Alberto Battistelli, Veronica De Micco, Marta Del Bianco, Greta Liuzzi, Stefano Moscatello, Roberta Paradiso, Simona Proietti, Youssef Rouphael, Stefania De Pascale

**Affiliations:** ^1^ Department of Agricultural Sciences, University of Naples Federico II, Portici, Naples, Italy; ^2^ Research Institute on Terrestrial Ecosystems (IRET), National Research Council of Italy (CNR), Porano, Terni, Italy; ^3^ Italian Space Agency, Rome, Italy

**Keywords:** anatomics, antioxidants, bioregenerative life support systems (BLSSs), *Brassica oleracea* var. *capitata* f. *sabauda*, light intensity, morpho-anatomical traits, *Raphanus raphanistrum* subsp. sativus, vapor pressure deficit (VPD)

## Abstract

During long-term manned missions to the Moon or Mars, the integration of astronauts’ diet with fresh food rich in functional compounds, like microgreens, could strengthen their physiological defenses against the oxidative stress induced by the exposure to space factors. Therefore, the development of targeted cultivation practices for microgreens in space is mandatory, since the cultivation in small, closed facilities may alter plant anatomy, physiology, and resource utilization with species-specific responses. Here, the combined effect of two vapor pressure deficit levels (VPD: 0.14 and 1.71 kPa) and two light intensities (150 and 300 µmol photons m^−2^ s^−1^ PPFD) on two species for microgreen production (*Brassica oleracea* var. *capitata* f. *sabauda* ‘Vertus’ and *Raphanus raphanistrum* subsp. *sativus* ‘Saxa’), was tested on biomass production per square meter, morpho-anatomical development, nutritional and nutraceutical properties. Microgreens were grown in fully controlled conditions under air temperature of 18/24°C, on coconut fiber mats, RGB light spectrum and 12 h photoperiod, till they reached the stage of first true leaves. At this stage microgreens were samples, for growth and morpho-anatomical analyses, and to investigate the biochemical composition in terms of ascorbic acid, phenols, anthocyanin, carotenoids, carbohydrates, as well as of anti-nutritional compounds, such as nitrate, sulfate, and phosphate. Major differences in growth were mostly driven by the species with ‘Saxa’ always presenting the highest fresh and dry weight as well as the highest elongation; however light intensity and VPDs influenced the anatomical development of microgreens, and the accumulation of ascorbic acid, carbohydrates, nitrate, and phosphate. Both ‘Saxa’ and ‘Vertus’ at low VPD (LV) and 150 PPFD increased the tissue thickness and synthetized high β-carotene and photosynthetic pigments. Moreover, ‘Vertus’ LV 150, produced the highest content of ascorbate, fundamental for nutritional properties in space environment. The differences among the treatments and their interaction suggested a relevant difference in resource use efficiency. In the light of the above, microgreens can be considered suitable for cultivation in limited-volume growth modules directly onboard, provided that all the environmental factors are combined and modulated according to the species requirements to enhance their growth and biomass production, and to achieve specific nutritional traits.

## 1 Introduction

The human settlement on Moon, Mars, or other celestial bodies as well as long-term living on transit space vehicles, are among the greatest challenges for mankind. However, to fulfill these purposes, many obstacles still need to be overcome, like the production of high nutritional food directly in space (Carillo et al., 2020; De Micco et al., 2021a; De Micco et al., 2021b). Indeed, one of the main constrains impacting the feasibility of long-term space missions is related to the huge mass and volume occupied by prepackaged, shelf-stable food, and to the unfeasibility of resupplying from Earth during manned interplanetary missions, lasting more than six months (Wheeler, 2003; Perchonok et al., 2012). Thus, a bio-regenerative system based on crop plants to produce food while emitting oxygen, absorbing carbon dioxide, and recycling gray water, will be a great asset to future space exploration (Poulet et al., 2016).

Growing plants in space requires the knowledge of their species-specific responses not only to the typical space constraints, such as altered gravity and ionizing radiation, but also to all the environmental factors that normally modulate plant performance on Earth. Environmental and cultivation factors (e.g., VPD, light intensity and quality, temperature, water availability, substrate), can alter plant growth and metabolic processes depending on the ontogenetic stage, and can have unexpected effects when acting in the confined environment and limited volume, typical of the space facilities (Amitrano et al., 2020a; De Micco et al., 2021a; De Pascale et al., 2021). Hence, in the upcoming years, to allow plant-based *in-situ* food production in space it would be mandatory to define the best ontogenetic specific environmental variable baselines and develop agronomical procedures to ensure an efficient food production, to meet the dietary needs of the crew members.

Microgreens are suitable for cultivation as food supplements because of their small size, low demand for photon flux, short cultivation cycle, high harvest index, and high nutritional potential (Kyriacou et al., 2017; El-Nakhel et al., 2021). Since the space environment poses significant risks to human health, primarily due to oxidative stress induced by ionizing radiation and microgravity (McPhee and Charles, 2009), microgreens consumption represents a valuable nutritional strategy to mitigate health risks during space travels (Kyriacou et al., 2017; Johnson, 2019; Teng et al., 2023). The extensive research on health promoting phytochemicals including vitamins, carotenoids, flavonoids and glucosinolates, has demonstrated the antioxidant potential of these compounds (Balsano and Alisi, 2009). Several works investigated the nutrition contribution of microgreens demonstrating that the daily intake of different nutraceuticals can be partially or fully obtained through microgreens supplementation (Ghoora et al., 2020; Renna et al., 2020).

Although microgreens are young plantlets, harvested at the most till the second true leaf stage, several studies demonstrated that the modulation of environmental and agronomic parameters influenced their response in terms of yield and phytochemical content (Samuolienė et al., 2013; Di Gioia et al., 2017; Lobiuc et al., 2017; Kyriacou et al., 2020).

Light is the source of energy for plant growth, and it is pivotal to many processes during plant life. In protected cultivation, the setting of an optimal light intensity is fundamental considering that light requirement may change among species and varieties (Poulet et al., 2014). A proper light modulation is also important for system design and efficiency in facilities dedicated to plant production under fully controlled environmental conditions, like vertical farming (Zabel et al., 2020; Kobayashi et al., 2022; Paglialunga et al., 2022). In the last decades, literature about plant-light interaction in controlled environment increased considerably, also by virtue of space related studies, after the invention of Light Emitting Diode (LED) technology (Avercheva et al., 2016; D’Aquino et al., 2022; Paradiso and Proietti, 2022). The modulation of light intensity can have positive effects on plants nutraceutical values (Proietti et al., 2013; Proietti et al., 2023), even when the intensity is high and could be perceived by plants as stress, inducing them to synthetize antioxidant compounds to mitigate ROS (reactive oxygen species) formation (Pattison et al., 2018; Modarelli et al., 2022). Excess of light can also affect the photosystems’ efficiency and inducing photo-inhibition mechanisms, other than causing the formation of a peculiar plant structure and changing the profiles and ratios of photosynthetic pigments (De Micco et al., 2023). It was recently demonstrated that it is possible to improve the antioxidant profile in several microgreen species by modulating the photoperiod, light intensity, and wavelength (Liu et al., 2022; Tantharapornrerk et al., 2023). Therefore, light modulation can be a powerful tool to control performances and quality of microgreens grown in space also considering interactions with other environmental variables.

Among environmental control needs, VPD regulation is one of the utmost challenges to face in indoor agriculture due to its dynamic interaction with crops in the soil-plant-atmosphere *continuum* (Leonardi et al., 2000; Amitrano et al., 2021a; Amitrano et al., 2021b). Recent studies clearly indicate that VPD impacts all physiological and biochemical processes related to transpiration, also affecting the morpho-anatomical development of crops in terms of leaf lamina tissues organization, stomatal and veins dimensions and densities, which in turn modify crops physiology in terms of gas-exchange and water use (Zhang et al., 2019; Amitrano et al., 2022). The influence of VPD on crops’ nutraceutical compounds has been way less studied compared to other environmental factors, but some research has agreed that high VPD levels (1.7 - 3 kPa) enhanced the bioactive value of some crops (tomato, cucumber, lettuce), increasing their ascorbic acid, β-carotene and polyphenolic content (Davey et al., 2000; Rosales et al., 2006; Bisbis et al., 2018). However, to the best of our knowledge, there is no information about the effect of different VPDs on microgreens production and nutritional potential nor on the combined effects on these parameters exerted by concomitant variations in light intensity and VPD.

Light and VPD are key aspects of the environmental control in Biorigenerative Life Support Systems (BLSSs) being crucial determinants of gas-exchange in leaves and particularly of transpiration. Moreover, the condensation of transpired water, driven by VPD and light conditions, is among the most demanding processes in controlled environment systems in terms on hardware and energy requirements, being even more difficult to control in space environment (Zabel et al., 2022). Moreover, as reported by Amitrano et al., 2020b on mung bean sprouts, both VPD and light have proven to change biomass allocation, antioxidant production, and morpho-functional traits already at the very early stage of development (e.g. germinated seeds, sprouts).

In this experiment, we cultivated two species (*Brassica oleracea* var. *capitata* f. *sabauda* ‘Vertus’ and *Raphanus raphanistrum* subsp. *sativus* ‘Saxa’) for microgreen production testing the combined effect of two vapor pressure deficits (VPD: 0.14 and 1.71 kPa) and two light intensities (150 and 300 µmol photons m^−2^ s^−1^). We analyzed microgreen morpho-anatomical development and biomass production to have an overview of their resource use-efficiency and growth. The innovative approach of the experiment is the strategy aiming to define system baselines, with an experimental protocol including interaction between light and VPD, with the target of achieving the nutritional quality expected for each species. To do so, we analyzed the nutritional and nutraceutical potential, in terms of ascorbic acid, phenols, anthocyanin, carotenoids, and carbohydrates, as well as the content of anti-nutritional compounds such as nitrate, sulfate and phosphate. Our results may contribute to the definition of the scientific requirements for the design of a flight apparatus for microgreen production in space.

## 2 Materials and methods

### 2.1 Plant material and experimental design

**Figure 1:**
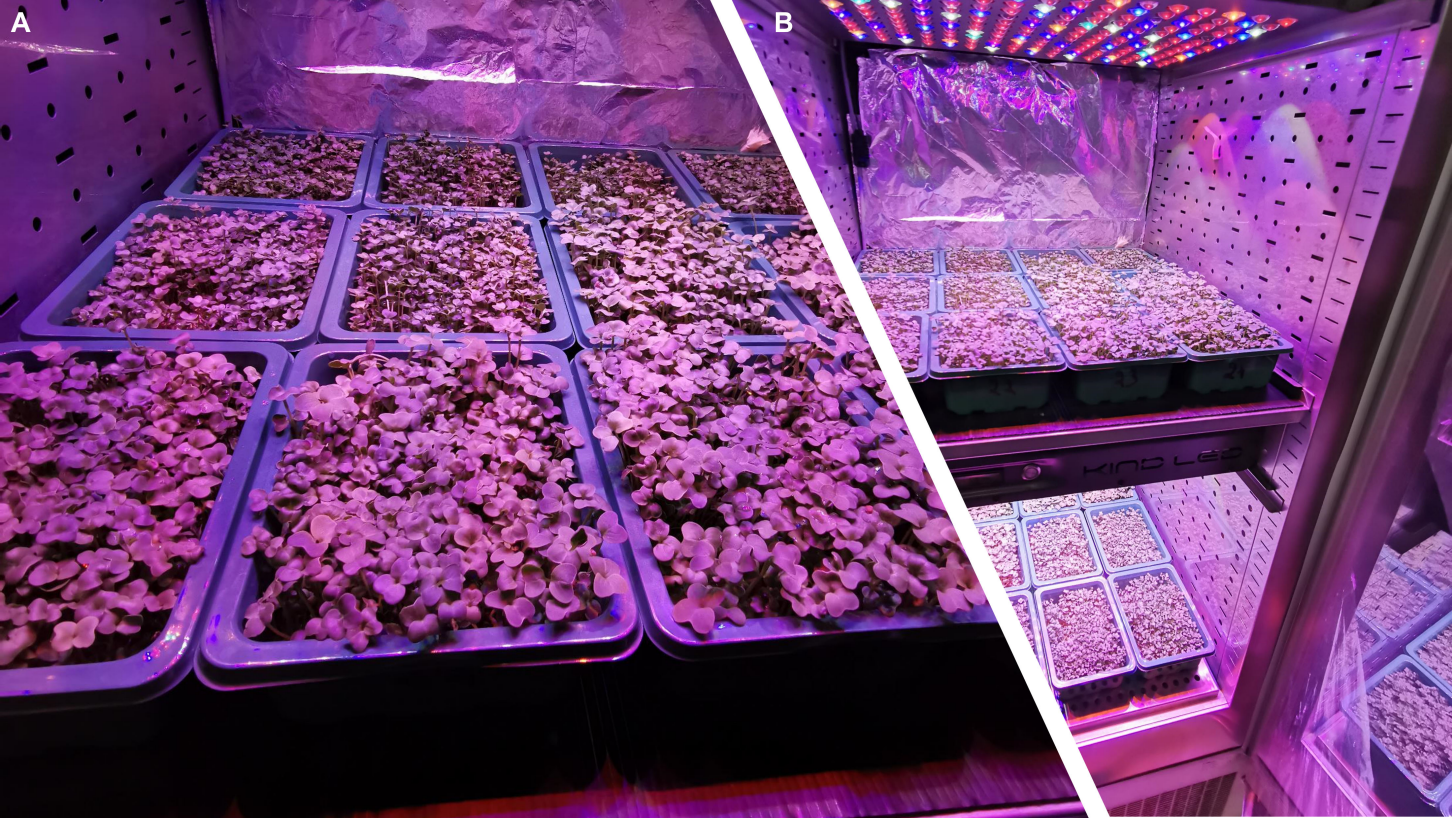
Different views **(A, B)** of the climatic growth chamber used for the two experiments on *Brassica oleracea* var. *capitata* f *sabauda* ‘Vertus’ and radish, *Raphanus raphanistrum* subsp. *sativus* ‘Saxa’ microgreens under Low VPD (LV) and High VPD (HV), at two light intensities (150 and 300 µmol photons m^−2^ s^−1^).

Two microgreen cultivars belonging to two different species were used: i) cabbage, *Brassica oleracea* var. *capitata* f. *sabauda* ‘Vertus’ and ii) radish, *Raphanus raphanistrum* subsp. *sativus* ‘Saxa’. For simplicity through the text and tables ‘Saxa’ and ‘Vertus’ will be indicated as species. The two species were previously selected in the first step of this project (please refer to paragraph 7: funding), through a rigorous selection process based on algorithms and cultivation trials (Izzo et al., 2023 Frontiers, under review) following the same approach reported in Aronne et al., 2020. Seeds were purchased from a local provider and experiments were run at the Department of Agricultural Sciences of the University of Naples Federico II (Naples, IT), in two consecutive cycles. Both cycles were conducted in a two-shelves growth chamber (KBP-6395F, Termaks, Bergen, Norway), equipped with a LED lighting panel (K5 Series XL750, Kind LED, Santa Rosa, CA, USA), with an emission wavelength in a range of 400-700 nm (**Figure 1**). The two cultivation cycles were identical in terms of light intensity, quality, photoperiod and air temperature, except for the VPD levels. The first cycle was performed under a VPD of 0.14 kPa (defined as Low VPD, LV) and the second under a VPD of 1.71 kPa (defined as High VPD, HV) at ambient CO_2_. The two VPDs were achieved keeping air temperature (T) at 24 ± 1°C during the day and 18 ± 2°C during the night and changing the RH accordingly. Both T and RH% were monitored inside the chamber using mini-sensors (Testo 174H, Testo, Germany), equipped with a data-logger collecting data every 15 minutes.7 g of seeds for ‘Saxa’ and 6 g for ‘Vertus’ were sown on coconut fiber mats into plastic trays (width 14 cm x lenght 19 cm x depht 6 cm), and fertigated with a quarter-strength modified Hoagland solution (in mM: 2.0 nitrate, 0.25 sulfur, 0.20 phosphorus, 0.62 potassium, 0.75 calcium, 0.17 magnesium, 0.25 ammonium; in μM: 20 iron, 9 manganese, 0.3 copper, 1.6 zinc, 20 boron, 0.3 molybdenum), with an electrical conductivity (EC) of 0.4 ± 0.1 dSm^−1^, and a pH of 6 ± 0.2. Two light intensities were set in the two chamber shelves, the first of 300 µmol m^−2^ s^−1^ photosynthetic photon flux density (PPFD) and the second of 150 µmol m^−2^ s^−1^, with a red (R), green (G), blue (B) spectrum of R45: G10: B45 (in % of the total emission), and a 12 h photoperiod.

Each treatment was replicated in 6 trays and daily rotation of the trays ensured a homogenous exposure to light and humidity. Microgreens were harvested at the appearance of the first two true leaves by cutting at the substrate level. Specifically, cultivation lasted 10 days in cabbage and 11 days in radish. At harvest, biometrical analyses were performed, and a sub-sample of 6 microgreens was stored in a fixative solution for anatomical analyses, while a second sub-sample of 10 g was rapidly frozen, lyophilized and stored at −80°C for biochemical analyses.

### 2.2 Colorimetric and biometric measurements

Just before harvesting, the color properties of the microgreen canopy were measured in three different positions of each tray by means of an 8 mm-aperture Minolta CR-400 Chroma Meter (Minolta Camera Co. Ltd., Osaka, Japan). The meter was calibrated with a standard white plate before measurements. Leaf chromaticity was performed following the *Commission Internationale de l’Eclairage* and expressed as: greenness (a*), yellowness (b*) and lightness (L*), used to calculate chroma (Chroma = (a*2+b*2)1/2) and Hue angle (H° = arctan (b*/a*).

After cutting, the whole harvested material was weighed to determine the fresh weight, expressed in kg m^−2^, 10 microgreens per tray were used to measure the microgreen length (ML). The dry weight (DW), expressed as g m^−2^, was obtained after drying the remaining material in a forced-air oven at 65°C until constant weight. Dry matter content (DM%) was also calculated and expressed as a percentage of fresh weight.

### 2.3 Plant anatomical investigation

For the anatomical investigation each microgreen leaf stored in the F.A.A. fixative solution (40% formaldehyde, glacial acetic acid, 50% ethanol, 5:5:90 by volume) was dissected to keep a portion of the median region of the lamina (about 5x5 mm), which was dehydrated in an ethanol series (from 50 to 95%), then embedded in the JB4 acrylic resin (Polysciences, USA). Thin cross sections (5 μm thick) were cut using a rotary microtome (pfm medical, Bio-optical, Germany) and mounted on a microscope slide, then stained with 0.025% toluidine blue (Feder and O’brien, 1968) and analyzed using a transmitted light microscope (BX51, Olympus, Germany) equipped with a digital camera (Olympus EP50, Olympus, Germany). Digital images were collected and analyzed through the Olympus cellSens 3.2 software, and thickness was measured in: i) the upper epidermis (UET), ii) the lower epidermis (LET), iii) the palisade parenchyma (PT), iv) the spongy parenchyma (ST), and v) the total leaf lamina (TLT) and expressed in µm. Moreover, the intercellular spaces (IS) were measured as the percentage of the area occupied by space among cells over a given surface of parenchyma. All the measurements were taken in 3 positions along the lamina, avoiding veins and damaged areas if present.

### 2.4 Ascorbate, anthocyanins and polyphenols content

For ascorbic acid (AsA) determination, 10 mg of freeze-dried powder were extracted using 2 mL of 3% metaphosphoric acid (MPA) in a glass-glass homogenizer at 4°C. The extract was then centrifuged at 16,000 g for 5 min at 4°C, then the supernatant was recovered and filtered through 0.2 µm PPII nylon filters (Whatman) before the quantification of AsA. For Dehydro-ascorbate (DHAs) quantification DHA in the filtered extract was reduced to AsA with the reducing agent Tris (2-carboxyethyl) phosphine (TCEP). 5 mmol L^−1^ TCEP was added in the filtered extract, which was then incubated for 30 min at 25°C under continuous shaking. After incubation the samples were injected into HPLC for the quantification of Total Ascorbic Acid (TAsA). The DHAs content was calculated as the difference between TAsA and AsA. The chromatographic quantification of ascorbic acid was performed as described by Chebrolu et al. (2012). Ascorbic acid content was determined using an HPLC U3000 system (Dionex™ ICS-5000; Thermo Fisher Scientific, Waltham, MA, United States). Separation was achieved using a Luna C18(2) (Phenomenex, Bologna, Italy) column with an isocratic mobile phase consisting of 0.010 mol L^−1^ of KH_2_PO_4_ pH 2.8. After a chromatographic run of 15 min (flow rate 0.7 mL min^−1^), AsA peak was detected at 254 nm using a UV/VIS detector (ThermoScientific ™ Dionex). Peaks processing was performed with the software Chromeleon 7.2 (ThermoScientific™ Dionex), and the quantifications were elaborated against the calibration curve of ascorbic acid standard (Supelco^®^, 595 N Harrison Rd, Bellefonte, PA 16823, USA).

Determination of Total Anthocyanins (TAcy) content was achieved by the extraction of 10 mg powder from freeze-dried material in 2 mL of 1% HCl in methanol for 1 hour at 65°C. The extract was clarified by centrifugation at 16,000 g for 5 min. The supernatant was then recovered, and the total anthocyanins content was determined spectrophotometrically, measuring the absorbance at 530 and 657 nm in order to correct the chlorophyll degradation products. The concentration was expressed as cyanidin-3-glucoside equivalent by using an extinction coefficient of 30,000 mol^−1^ cm^−1^. Total phenolic content (TPoly) was determined following Usenik et al. (2008). Extraction was conducted with 10 mg of the freeze-dried powder in 2 mL of 100% methanol. Supernatant was recovered after centrifugation at 16,000 g for 5 min and used to measure spectrophotometrically the absorbance at 765 nm. TPoly was then calculated by relating the absorbance of each sample with the calibration curve of gallic acid (Supelco^®^, 595 N Harrison Rd, Bellefonte, PA 16823, USA).

### 2.5 Photosynthetic pigments and carbohydrates content

For pigment determinations, 10 mg of freeze-dried microgreen powder was extracted in a glass-glass homogenizer, with 2 mL 100% acetone at 4°C, under dark conditions. The extracts were centrifuged at 16,000 g for 5 min at 4°C and filtered through a 0.2 μm nylon PPII syringe disposable filter; 15 μl of the filtered extract were used to assay the concentration of neoxanthin (Neo), violaxanthin (Vio), luteine (Lut), β-carotene (β-car), chlorophyll a and b (Chl a+b), using a HPLC U3000 system (Dionex™ ICS-5000; Thermo Fisher Scientific, Waltham, MA, United States), equipped with a Luna C18(2) (Phenomenex, Bologna, Italy) analytical column (5 μm, 250 mm × 4.6 mm) and a related guard column (Phenomenex, Bologna, Italy), maintained at 30°C. The separation run lasted 22 min at a flow rate of 1 mL min^−1^. From 0 to 4 min, the mobile phase was composed of solution A: 1.75% water, 1.75% methanol, 1.75% dichloromethane, and 94.75% acetonitrile; from 4.1 to 18 min the mobile phase was composed of solution B: 50% acetonitrile and 50% diethyl acetate. Final re-equilibration of 4 min was conducted with solution A. The autosampler temperature was set at 4°C, the UV detector wavelength was 440 nm, and the concentration of all the pigments was quantified against standard curves (Supelco^®^, 595 N Harrison Rd, Bellefonte, PA 16823, USA) as reported in Žnidarčič et al. (2011).

The extraction of total non-structural carbohydrates (TNsc) was conducted using 10 mg of the freeze-dried powder material in 1 mL of 80% ethanol at 80°C for 45 min, under continuous shaking. The extract was centrifuged at 16,000 g for 5 min, then soluble sugars (glucose, fructose, and sucrose) were recovered in the supernatant and starch in the pellet. The measurement of total soluble sugars (TSolub) was performed by spectrophotometric coupled enzymatic assay as described by Scartazza et al. (2017). The sugar assays were performed in dual-wavelength mode (340-405 nm) in an Elisa plate reader (Spectrostar Nano, BMG Labtech, Germany). After separation, the supernatant containing soluble sugars, was immediately analyzed for soluble sugar content, or stored at −20°C until analysis. The pellet was washed four times with a 50 mM Na Acetate buffer (pH 4.5), suspended in 1 mL of the same buffer and autoclaved at 120°C for 45 min to solubilize the starch. After autoclaving, the pellets were incubated at 50°C for 1 h with amyloglucosidase (70 U) and α-amylase (4U) for the complete starch hydrolysis. The determination of starch was conducted by measuring the glucose, produced by the hydrolysis, as described before for the soluble sugars content.

### 2.6 Anions analysis

The extraction of inorganic anions (nitrate, sulfate, and phosphate) was achieved using 10 mg freeze-dried powder in water at 80°C for 45 min, under continuous shaking. After the extraction, the sample was centrifuged at 16,000 g for 5 min and the supernatant was recovered and filtrated using a 0.2 μm nylon PPII syringe filter, before the injection in a ion chromatography system (DionexTM ICS-5000; Thermo Fisher Scientific, Waltham, MA, United States), equipped with a conductivity detector, an analytical IonPac AS11-HC column (4 × 250mm) (Thermo Fisher Scientific) and a related guard column and IonPac Anion Trap Column (ATC)^-1^ (Thermo Fisher Scientific). The chromatographic system was coupled with an ERSTM 500 Electrolytically Regenerated Suppressor, (DionexTM ICS- 5000; Thermo Fisher Scientific) to suppress unwanted ionic interference in the analysis. The runs were carried out at a temperature of 30°C and a flow rate of 1 mL min^−1^. A sodium hydroxide stepped gradient was used as the mobile phase as in Proietti et al. (2019). The electrical signal was integrated into micro-Siemens (μS). The eluents and the inorganic anion standard solutions were prepared using HPLC-grade reagents (Merck KGaA, Darmstadt, Germany). The chromatographic system control, data acquisition and processing were performed by the software Chromeleon 7.2 (ThermoScientific™ Dionex).

### 2.7 Statistical analyses

To test the influence of the three independent factors, i) Species, ii) VPD, iii) Light intensity, on the dependent variables, a three-way analysis of variance (ANOVA) was performed using the IBM SPSS Statistics software (SPSS, Chicago, IL, USA). When the interactions were significant, a one-way ANOVA was performed, separating the values according to Tukey test with a p value ≤ 0.05. Moreover, a multivariate analysis was performed as principal component analysis (PCA) on all the calculated traits (biometric, anatomical, and biochemical traits), using the software Past3 (Natural History Museum, University of Oslo, Norway). Variables used as input for multivariate analysis were standardized to zero mean and unit variance.

## 3 Results

### 3.1 Growth analysis

**Figure 2:**
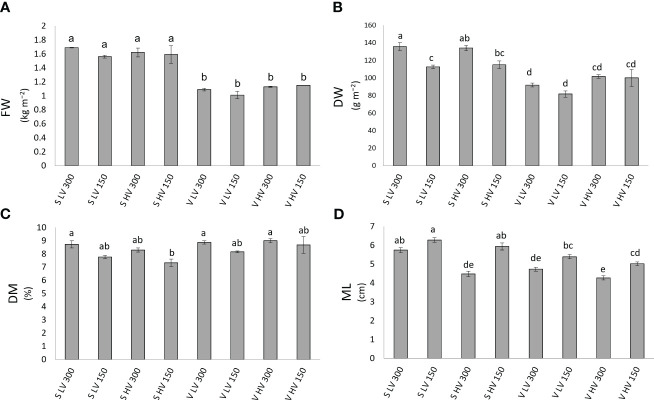
Fresh weight (FW, **(A)**, dry weight (DW, **(B)**, dry matter content (DM%, **(C)** and microgreens length (ML, **(D)**, of ‘Saxa’ and ‘Vertus’ microgreens grown under Low VPD (LV) and High VPD (HV) at the two light intensities (150 and 300 µmol photons m^−2^ s^−1^). All data are expressed as mean ± standard errors. Different letters correspond to statistically significant differences according to Tukey test (p ≤ 0.05).

The main factors of this investigation (species, VPD and light) had a significant effect, alone and in interaction, on microgreen growth depending on the specific parameter (**Table 1**, **Figure 2**). Particularly, the species alone elicited a significant effect (p ≤ 0.001) on the fresh weight (FW), dry weight (DW) and microgreen length (ML), always showing higher values in ‘Saxa’ compared to ‘Vertus’. Differently, the VPD had a significant effect (p ≤ 0.001) only on ML where at lowest values (LV) corresponded a higher elongation. Light elicited significant effects (p ≤ 0.01) on DW and DM%, with increments of both parameters at 300 PPFD, and on ML (p ≤ 0.001) with decreases in elongation at 300 PPFD. The interaction among the three factors (SxVxL) showed a significance of p ≤ 0.01 for DW and ML, but it was non-significant for FW and DM%.

**Table 1:** Analysis of variance and means comparison for growth-related traits of Fresh Weight (FW), Dry Weight (DW), Dry Matter Content (DM) and microgreen length (ML) of ‘Saxa’ and ‘Vertus’ microgreens grown under Low VPD (LV) and High VPD (HV), under two light intensities: 150 and 300 PPFD (µmol photons m^−2^ s^−1^).

	FW (kg m^−2^)	DW (g m^−2^)	DM (%)	ML (cm)
Species				
Saxa (S)	1.36 ± 0.43a	124 ± 13.5a	8.62 ± 0.73	5.62 ± 1.03a
Vertus (V)	1.09 ± 0.11b	91.4 ± 15.6b	8.67 ± 0.82	4.86 ± 0.69b
VPD				
Low (LV)	1.08 ± 0.32	102 ± 24.4	8.38 ± 0.58	5.53 ± 0.83a
High (HV)	1.07 ± 0.29	113 ± 18.8	8.32 ± 1.04	4.93 ± 0.98b
Light				
300 PPFD	1.35 ± 0.26	113 ± 24.2a	8.71 ± 0.50a	4.82 ± 0.86b
150 PPFD	1.32 ± 0.37	102 ± 18.6b	7.98 ± 0.95b	5.66 ± 0.85a
Significance				
Species	***	***	NS	***
VPD	NS	NS	NS	***
Light	NS	**	**	***
SxVxL	NS	**	*	**

NS, *, **, *** stands for non-significant or significant at p ≤ 0.05, p ≤ 0.01, p ≤ 0.001. Different letters correspond to significant differences according to Tukey test.

Concerning the DW (**Figure 2B**), highest values were found in S LV 300 and S HV 300, in turn higher than S LV 150 and S HV 150, followed by V HV 150 and 300; lowest values were observed in V LV 150 and 300. For the ML (**Figure 2D**), highest values were found in S LV 150 and 300 and S HV 150, followed by V LV 150 and V HV 150, which were in turn higher than S HV 300 and V LV 300; lowest values were found in V HV 300.

### 3.2 Colorimetry analysis

**Table 2** shows the results from colorimetry analysis. Here, the effect of the species alone was significant for all the parameters analyzed (p ≤ 0.001), except for the Hue, always showing higher values in ‘Saxa’ compared to ‘Vertus’. Differently, the VPD had significant effects only on brightness (b*) (p ≤ 0.05) with increments in the parameter at HV and on Hue angle (p ≤ 0.001) with decreases in the parameter at HV. The light intensity was significant for b*, chroma and Hue (p ≤ 0.001), and lightness (L*) (p ≤ 0.01). The highest light intensity (300 PPFD) determined higher values of b*, L* and chroma but lower Hue. Here, the interaction SxVxL was significant, only for the Hue value (p ≤ 0.001) which showed higher values in S HV 150 and V LV 150, followed by S LV at both 150 and 300, which were in turn higher than V HV 150 and V LV 300. Lowest values were found in S HV 300 and V HV 300.

**Table 2:** Analysis of variance and means comparison for colorimetry-related traits of greenness (a*), yellowness (b*), lightness (L*), Chroma and Hue angle of ‘Saxa’ and ‘Vertus’ microgreens grown under Low VPD (LV) and High VPD (HV) at the two light intensities (150 and 300 µmol photons m^−2^ s^−1^).

	a*	b*	L*	Chroma	Hue
Species					
Saxa (S)	-13.3 ± 1.24a	20.68 ± 2.34a	36.6 ± 5.07a	24.6 ± 2.53a	123± 1.85
Vertus (V)	-11.7 ± 0.86b	18.25 ± 1.73b	34.5 ± 3.55b	21.7 ± 1.83b	123 ± 1.67
VPD					
Low (LV)	-12.3 ± 1.37	18.97 ± 2.46b	35.4 ± 4.76	22.7 ± 2.75	123 ± 1.56a
High (HV)	-12.4 ± 1.24	19.73 ± 2.12a	35.3 ± 3.89	23.4 ± 2.34	122 ± 1.87b
Light					
300 PPFD	-12.5 ± 1.43	20.15 ± 2.01a	36.2 ± 4.36a	23.7 ± 2.21a	122 ± 1.62b
150 PPFD	-12.3 ± 1.14	19.30 ± 2.35b	34.7 ± 4.33b	22.4 ± 2.73b	123 ± 1.53a
Interaction					
S LV 300	-13.5 ± 0.14	20.9 ± 0.35	38.7 ± 0.56	24.9 ± 0.36	123 ± 0.29ab
S LV 150	-13.2 ± 0.27	20.2 ± 0.47	35.0 ± 1.09	24.2 ± 0.53	123 ± 0.26ab
S HV 300	-13.1 ± 0.13	21.5 ± 0.29	37.8 ± 0.69	25.2 ± 0.29	121 ± 0.27d
S HV 150	-13.5 ± 0.22	20.1 ± 0.37	35.1 ± 0.71	24.3 ± 0.41	124 ± 0.26a
V LV 300	-11.8 ± 0.14	18.9 ± 0.25	34.3 ± 0.74	22.3 ± 0.28	122 ± 0.20cd
V LV 150	-11.6 ± 0.16	17.4 ± 0.26	34.7 ± 0.57	20.9 ± 0.30	124 ± 0.19a
V HV 300	-11.7 ± 0.13	19.4 ± 0.23	34.3 ± 0.55	22.6 ± 0.25	121 ± 0.22d
V HV 150	-11.6 ± 0.12	18.1 ± 0.27	34.3 ± 0.45	21.5 ± 0.27	123 ± 0.28bc
Significance					
Species	***	***	***	***	NS
VPD	NS	*	NS	NS	***
Light	NS	***	**	***	***
SxVxL	NS	NS	NS	NS	***

NS, *, **, *** stands for non-significant or significant at p ≤ 0.05, p ≤ 0.01, p ≤ 0.001. Different letters correspond to significant differences according to Tukey test.

### 3.3 Anatomical analysis

**Figure 3:**
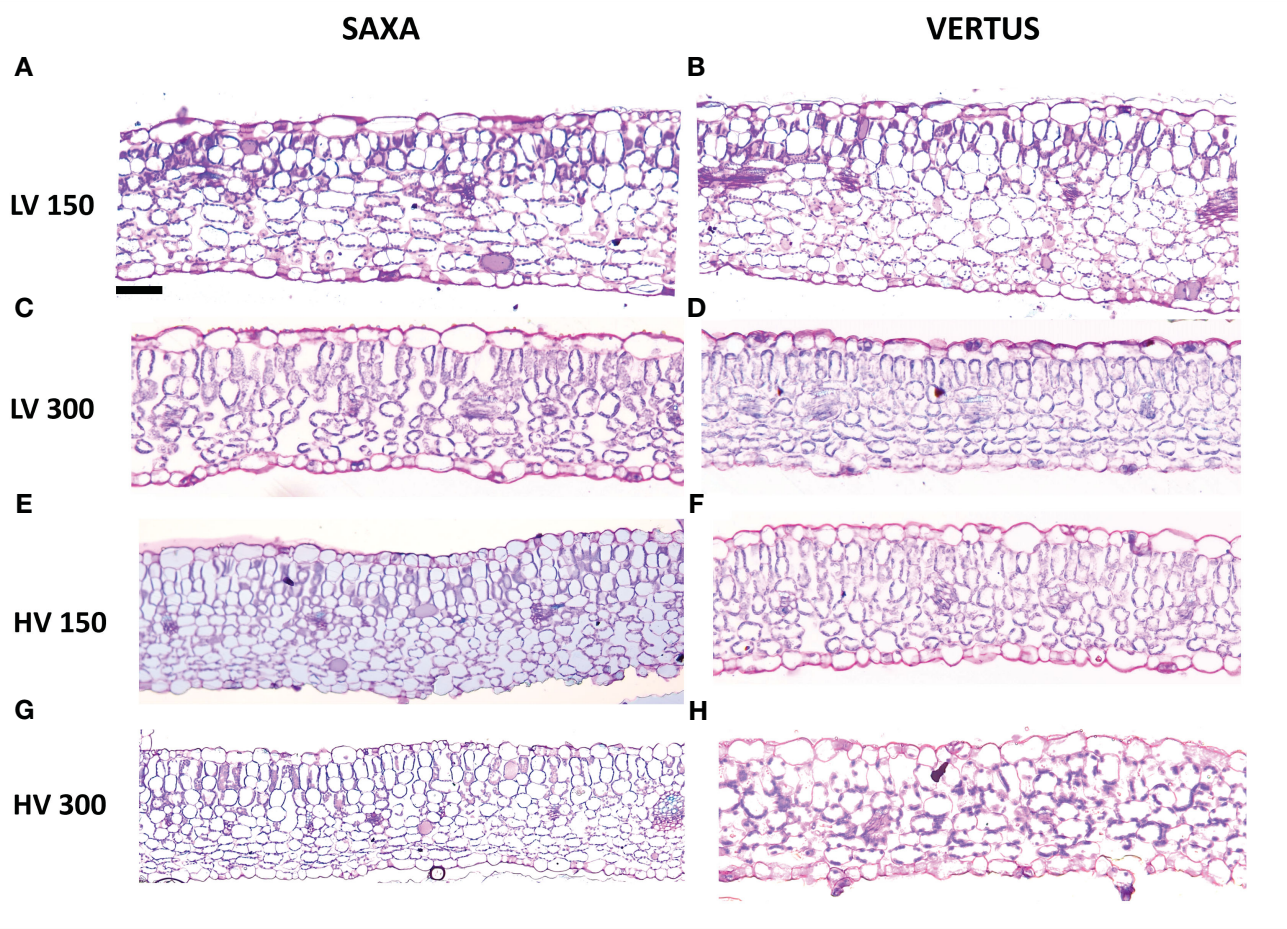
Light microscopy views of leaf lamina cross sections randomly chosen among the many shots of ‘Saxa’ **(A, C, E, G)** and ‘Vertus’ **(B, D, F, H)** microgreens under low VPD (LV, **(A–D)** and high VPD (HV, **(E–H)** at the two light intensities (150 PPFD, **(A, B, E, F)** and 300 PPFD, **(C, D, G, H)**. Images are at the same magnification, scale bar = 50μm.

All the treatments allowed the formation of an ordinary mesophyll structure (**Figure 3**), however there were significant differences in the parameters analyzed among treatments (**Table 3**). The main factors had a significant effect, alone and in interaction, depending on the specific parameter. The Species elicited significant differences except for the lower epidermis thickness (LET), always with higher values in ‘Saxa’ but with a different degree of significance: p value ≤0.001 for palisade thickness (PT), spongy thickness (ST), and intercellular space percentage (IS), and p ≤ 0.01 for the upper epidermis thickness (UET) and total lamina thickness (TLT). The VPD significantly influenced PT (p ≤ 0.01), ST (p ≤ 0.05), TLT (p ≤ 0.001) with increments at LV and IS (p ≤ 0.001) with reduction at LV. Differently, light intensity significantly influenced PT (p ≤ 0.01) with increments at 300 PPFD and TLT (p ≤ 0.01), ST, and IS (p ≤ 0.001) with increments at 150 PPFD.

**Table 3:** Analysis of variance and means comparison for anatomical-related traits of Upper Epidermis Thickness (UET), Palisade parenchyma Thickness (PT), Spongy parenchyma Thickness (ST), Lower Epidermis Thickness (LET), Total Lamina Thickness (TLT) and the percentage of Intercellular Spaces (IS) of ‘Saxa’ and ‘Vertus’ microgreens grown under Low VPD (LV) and High VPD (HV) at the two light intensities (150 and 300 µmol photons m^−2^ s^−1^).

	UET (µm)	PT (µm)	ST (µm)	LET (µm)	TLT (µm)	IS (%)
Species						
Saxa (S)	23.1 ± 5.14a	110 ± 23.4a	187 ± 55.2a	20.2 ± 5.40	340 ± 63.4a	37.6 ± 0.71a
Vertus (V)	21.1 ± 5.12b	97.3 ± 20.0b	157 ± 36.8b	19.8 ± 4.30	295 ± 41.5b	28.4 ± 0.62b
VPD						
Low (LV)	22.2 ± 5.11	107 ± 23.6a	180 ± 61.1a	20.6 ± 5.56a	331 ± 66.1a	30.4 ± 1.14b
High (HV)	22.1 ± 5.32	101 ± 21.5b	165 ± 34.8b	19.5 ± 4.15b	308 ± 48.4b	35.8 ± 1.12a
Light						
300 PPFD	22.2 ± 5.76	120 ± 25.3a	150 ± 36.8b	19.8 ± 5.48	209 ± 10.7b	21.6 ± 0.53b
150 PPFD	22.1 ± 4.76	100 ± 19.8b	190 ± 51.4a	20.2 ± 4.39	301 ± 53.8a	41.3 ± 0.53a
Interaction						
S LV 300	24.3 ± 1.09a	120 ± 4.01a	162 ± 5.58b	21.5 ± 1.41	328 ± 8.67b	24.4 ± 0.18f
S LV 150	21.1 ± 0.67ab	107 ± 3.83abc	243 ± 11.2a	19.8 ± 0.90	391 ± 12.5a	43.8 ± 0.32b
S HV 300	22.8 ± 0.89a	104 ± 5.61bcd	159 ± 6.32bc	18.9 ± 0.82	305 ± 10.9b	27.5 ± 0.56e
S HV 150	24.4 ± 0.91a	111 ± 2.41abc	172 ± 3.11b	20.6 ± 0.69	199 ± 3.93c	49.6 ± 0.13a
V LV 300	22.8 ± 1.19a	118 ± 3.21ab	128 ± 4.13c	21.2 ± 0.88	291 ± 5.67bc	14.4 ± 0.18h
V LV 150	20.9 ± 0.77ab	84.5 ± 2.44e	166 ± 4.23b	20.1 ± 0.90	292 ± 5.36bc	34.5 ± 0.22d
V HV 300	18.1 ± 0.92b	90.6 ± 3.48de	150 ± 10.2bc	17.8 ± 0.91	277 ± 12.9bc	19.5 ± 0.15g
V HV 150	21.9 ± 0.75ab	95.5 ± 2.65cde	174 ± 5.14b	20.1 ± 0.54	311 ± 5.48b	39.4 ± 0.17c
Significance						
Species	**	***	***	NS	**	***
VPD	NS	**	*	*	***	***
Light	NS	**	***	NS	**	***
SxVxL	*	*	**	NS	***	**

NS, *, **, *** stands for non-significant or significant at p ≤ 0.05, p ≤ 0.01, p ≤ 0.001. Different letters correspond to significant differences according to Tukey test.

The interaction among the main factors (SxVxL) significantly influenced all parameters except for LET, with UET and PT (p ≤ 0.05), ST and IS (p ≤ 0.01) and TLT (p ≤ 0.001). ‘Saxa’ developed thicker leaves with more intercellular spaces. Overall, microgreens at LV resulted thicker with less IS% compared to HV and microgreens at 150 PPFD enhanced their total thickness, the thickness of the spongy parenchyma and IS % compared to microgreens grown at 300 PPFD, which however had a thicker palisade tissue.

### 3.4 Ascorbate, anthocyanins and phenolic content

The effect of the three main factors (species, light intensity and VPD) alone and in interaction on dehydroascorbic acid (DHAs), ascorbic acid (Asa), total ascorbic acid (TAsA), total anthocyanins (TAcy), and total phenolic content (TPoly) are reported in **Table 4**. ‘Vertus’ microgreens showed significantly higher values than ‘Saxa’ for all variables except for TAcy. The effect of VPD was significant only in DHAs and AsA. The LV treatment increased the content of DHAs and decreased that of AsA. The two light intensity levels differentially modulated the accumulation of AsA (p ≤ 0.01) and Tot AsA (p ≤ 0.001), always with higher content at 300 PPFD than at 150 PPFD.

**Table 4:** Analysis of variance and means comparison for dehydro-ascorbate (DHAs), ascorbate (AsA), total ascorbate (TAsA), total anthocyanins (TAcy) and total polyphenols (TPoly) content of ‘Saxa’ and ‘Vertus’ microgreen grown under Low VPD (LV) and High VPD (HV) at the two light intensities: 150 and 300 PPFD (µmol photons m^−2^ s^−1^).

	DHAs	AsA	TAsA	TAcy	TPoly
	(mg 100g FW^-1^)	(mg 100g FW^-1^)	(mg 100g FW^-1^)	(mg 100g FW^-1^)	(mg 100g FW^-1^)
Species					
Saxa (S)	16.6 ± 2.13b	13.1 ± 1.81b	29.7 ± 2.48b	6.04 ± 0.66	145 ± 8.57b
Vertus (V)	37.2 ± 2.76a	24.5 ± 3.06a	61.6 ± 2.06a	5.59 ± 0.33	193 ± 8.94a
VPD					
Low (LV)	31.4 ± 3.9a	15.7 ± 2.09b	47.1 ± 4.97	6.46 ± 0.59	172.± 10.26
High (HV)	22.4 ± 3.53b	21.8 ± 3.53a	44.2 ± 5.62	5.21 ± 0.38	166 ± 12.28
Light					
300 PPFD	27.8 ± 3.5a	23.1 ± 3.46a	50.9 ± 5.01a	6.37 ± 0.32	175 ± 10.52
150 PPFD	25.9 ± 4.36a	14.4 ± 1.77b	40.4 ± 5.14b	5.30 ± 0.64	163 ± 11.82
Interaction					
S LV 300	21.7 ± 1.27bc	16.5 ± 5.12	38.3 ± 4.77	6.35 ± 0.26	151 ± 2.2
S LV 150	18.6 ± 5.54c	8.95 ± 2.35	27.6 ± 3.18	7.32 ± 2.45	162 ± 34.2
S HV 300	18.2 ± 1.76c	13.6 ± 1.47	31.8 ± 3.12	6.68 ± 1.08	140 ± 6.28
S HV 150	8.02 ± 3.36c	13.1 ± 4.88	21.2 ± 3.98	4.41 ± 0.12	127 ± 7.45
V LV 300	46.4 ± 3.97a	22.4 ± 3.87	68.7 ± 1.02	6.95 ± 0.73	185 ± 11.6
V LV 150	38.9 ± 4.36ab	14.9 ± 2.03	53.9 ± 4.75	5.23 ± 0.09	190 ± 22.1
V HV 300	25.3 ± 2.92bc	40.0 ± 3.21	64.9 ± 0.21	5.54 ± 0.25	225 ± 5.50
V HV 150	38.3 ± 3.06ab	20.6 ± 0.91	58.8 ± 2.46	4.22 ± 0.06	171 ± 17.5
Significance					
Species	***	***	***	NS	***
VPD	**	*	NS	NS	NS
Light	NS	**	***	NS	NS
S x V x L	*	NS	NS	NS	NS

NS, *, **, *** stands for non-significant or significant at p ≤ 0.05, p ≤ 0.01, p ≤ 0.001. Different letters correspond to significant differences according to Tukey test.

The interaction among the main factors (SxVxL) only influenced the DHAs content, determining the highest value in ‘Vertus’ under 300 PPFD and Low VPD (V LV 300) (p ≤ 0.05). The TAcy content was not significantly influenced by any of the experimental factors.

### 3.5 Photosynthetic pigment and carbohydrates content

The pigments accumulation was mainly driven by the species, determining significant differences between ‘Saxa’ and ‘Vertus’. Indeed, ‘Vertus’ has a higher content of neoxanthin (p ≤ 0.05), lutein (p ≤ 0.01), chlorophylls a+b (p ≤ 0.05) and β-carotene (p ≤ 0.01) compared to ‘Saxa’ (**Table 5**). A different level of accumulation of pigments was found for neoxanthin (p ≤ 0.001), lutein (p ≤ 0.05) and β-carotene (p ≤ 0.05) in response to the VPD, with a higher content of these compounds at LV. Conversely, the different light intensities as well as the interaction among factors did not determine significant variations for any of the pigments analyzed. Concerning the starch, total soluble non-structural carbohydrates (TSolub), and total non-structural carbohydrates (TNsc) higher values were found in ‘Vertus’ microgreens (p ≤ 0.001) (**Table 5**). The VPD was statistically significant only in starch (p ≤ 0.05), with higher values under LV, while the light intensity resulted significant in all the parameters (p ≤ 0.001), always with increments at 300 PPFD. The three factors interaction resulted to influence only the accumulation of starch (p ≤ 0.01), that was two-fold higher in the combination V HV 300 compared to V LV 300 and over 10 times greater compared to the averages values of the other experimental conditions.

**Table 5:** Analysis of variance and means comparison for Neoxanthin (Neo), Violaxanthin (Vio), Lutein (Lut), Chlorophylls (Chl a+b), β-caroten (β-car), Starch, total soluble sugars (TSolub), Total Non-Structural carbohydrates (TNsc) content of ‘Saxa’ and ‘Vertus’ microgreens grown under Low VPD (LV) and High VPD (HV) at the two light intensities:150 and 300 PPFD (µmol photons m^−2^ s^−1^).

	Neo	Vio	Lut	Chl a+b	β-Car	Starch	TSolub	TNsc
	(mg 100g FW^-1^)	(mg 100g FW^-1^)	(mg 100g FW^-1^)	(mg 100g FW^-1^)	(mg 100g FW^-1^)	(mg 100g FW^-1^)	(mg 100g FW^-1^)	(mg 100g FW^-1^)
Species								
Saxa (S)	1.40 ± 0.10b	27.5 ± 2.59	4.98 ± 0.35b	64.3 ± 5.26b	3.64 ± 0.25b	54.7 ± 9.28b	463.9 ± 46.7 b	518.6 ± 54.9b
Vertus (V)	1.69 ± 0.134a	22.9 ± 1.61	6.60 ± 0.37a	82.0 ± 5.14a	4.60 ± 0.22a	258 ± 72.3a	1127 ± 70.7a	1386.3 ± 128.9a
VPD								
Low (LV)	1.80 ± 0.12a	27.9 ± 2.54	6.27 ± 0.44a	79.0 ± 6.21	4.44 ± 0.31a	129 ± 35.6a	831.9 ± 111.7	960.8 ± 139.8
High (HV)	1.28 ± 0.83b	22.5 ± 1.55	5.32 ± 0.37b	67.3 ± 4.85	3.80 ± 0.20b	184 ± 76.1b	759.7 ± 120.3	944.0 ± 185.2
Light								
300 PPFD	1.50 ± 0.11	25.2 ± 1.0	5.76 ± 0.39	70.9 ± 4.9	4.05 ± 0.25	271 ± 68.9a	954.6 ± 116.4a	1226.3 ± 174.2a
150 PPFD	1.58 ± 0.14	25.2 ± 3.04	5.82 ± 0.47	75.4 ± 6.54	4.20 ± 0.30	41.6 ± 6.44b	637.0 ± 95.3b	678.7 ± 99.4b
Interaction								
S LV 300	1.49 ± 0.15	25.1 ± 3.25	4.84 ± 0.61	58.1 ± 8.35	3.39 ± 0.33	94.4 ± 4.81c	628.4 ± 46.2	722.8 ± 41.5
S LV 150	1.67 ± 0.32	35.3 ± 9.43	6.12 ± 1.21	81.3 ± 17.91	4.47 ± 0.89	42.8 ± 10.9c	402.1 ± 83.7	444.9 ± 90.7
S HV 300	1.03 ± 0.17	24.6 ± 1.07	4.34 ± 0.03	54.4 ± 1.87	3.14 ± 0.17	64.2 ± 11.4c	533.5 ± 56.6	597.7 ± 67.9
S HV 150	1.4 ± 0.07	24.7 ± 2.62	4.64 ± 0.12	63.3 ± 1.09	3.57 ± 0.1	17.3 ± 4.07c	291.9 ± 54.5	309.2 ± 57.0
V LV 300	1.93 ± 0.05	26.3 ± 1.21	6.65 ± 0.33	82.2 ± 5.03	4.80 ± 0.02	319 ± 53.6b	1355.6 ± 87.79	1674.9 ± 34.4
V LV 150	2.12 ± 0.28	25.0 ± 2.26	7.47 ± 0.8	94.4 ± 9.48	5.11 ± 0.63	59.3 ± 5.65c	941.5 ± 42.1	1000.7 ± 42.6
V HV 300	1.57 ± 0.13	24.7 ± 2.83	7.23 ± 0.29	88.9 ± 2.23	4.85 ± 0.14	608 ± 79.9a	1300.9 ± 52.4	1909.5 ± 45.6
V HV 150	1.13 ± 0.12	15.8 ± 2.81	5.06 ± 0.67	62.6 ± 3.19	3.63 ± 0.24	47.2 ± 16.6c	912.7 ± 128.2	959.9 ± 130.6
Significance								
Species	*	NS	**	*	**	***	***	***
VPD	***	NS	*	NS	*	*	NS	NS
Light	NS	NS	NS	NS	NS	***	***	***
S x V x L	NS	NS	NS	NS	NS	**	NS	NS

NS, *, **, *** stands for non-significant or significant at p ≤ 0.05, p ≤ 0.01, p ≤ 0.001. Different letters correspond to significant differences according to Tukey test.

### 3.6 Anions content

The anions analysis revealed that the two species differently accumulated nitrate, sulfate, and phosphate (**Table 6**), with ‘Saxa’ reaching a content of nitrate (p ≤ 0.001) and phosphate (p ≤ 0.01) about two-fold higher compared to ‘Vertus’. On the other hand, ‘Vertus’ accumulated more sulfate (p ≤ 0.001). The effect of VPD resulted statistically significant only for the accumulation of nitrate (p ≤ 0.05) where the LV led to 52% increment compared to HV. Differently, the light intensity alone was never significant as well as the interaction among factors.

**Table 6:** Analysis of variance and means comparison for nitrate, sulfate, phosphate content of ‘Saxa’ and ‘Vertus’ microgreens grown under Low VPD (LV) and High VPD (HV) at the two light intensities (150 and 300 µmol photons m^−2^ s^−1^).

	Nitrate	Sulfate	Phosphate
	(mg 100g FW^-1^)	(mg 100g FW^-1^)	(mg 100g FW^-1^)
Species			
Saxa (S)	74.9 ± 6.84a	12.4 ± 1.19b	32.7 ± 4.5a
Vertus (V)	36.1 ± 8.74b	23.6 ± 1.31a	17.9 ± 2.13b
VPD			
Low (LV)	66.5 ± 9.96a	18.4 ± 2.38	28.2 ± 5.08
High (HV)	43.7 ± 8.71b	17.6 ± 1.79	22.4 ± 2.74
Light			
300 PPFD	50.3 ± 8.52	19.3 ± 2.1	21.0 ± 2.47
150 PPFD	58.6 ± 10.8	16.8 ± 2.05	29.6 ± 5.04
Interaction			
S LV 300	58.7 ± 8.49abc	11.9 ± 1.48	25.5 ± 5.78
S LV 150	94.6 ± 18.3a	11.3 ± 3.19	48.5 ± 13.2
S HV 300	57.5 ± 2.64abc	14.8 ± 2.85	21.6 ± 3.17
S HV 150	83.5 ± 4.90ab	11.5 ± 2.59	35.4 ± 3.16
V LV 300	66.6 ± 26.1abc	28.2 ± 0.62	17.2 ± 7.78
V LV 150	43.7 ± 10.2abc	22.3 ± 3.16	21.5 ± 2.3
V HV 300	21.3 ± 4.03bc	22.0 ± 2.28	19.8 ± 3.54
V HV 150	12.6 ± 0.48c	22.1 ± 2.81	12.9 ± 0.97
Significance			
Species	***	***	**
VPD	*	NS	NS
Light	NS	NS	NS
S x V x L	*	NS	NS

NS, *, **, *** stands for non-significant or significant at p ≤ 0.05, p ≤ 0.01, p ≤ 0.001. Different letters correspond to significant differences according to Tukey test.

### 3.7 Principal component analysis

**Figure 4:**
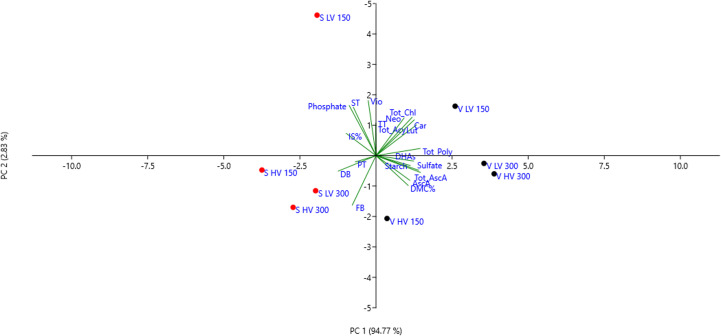
Principal component analysis (PCA) loading plot and scores of biometric, anatomical, and biochemical traits of ‘Saxa’ and ‘Vertus’ microgreens under Low VPD (LV) and High VPD (HV) at the two light intensities (150 and 300 µmol photons m^−2^ s^−1^).

The PCA scatter plot reported in **Figure 4** shows that the first two components explained a 97.6% of the total variance, with PC1 explaining most of the total variance (94.8%) and PC2 the remaining 2.8%. The first component (PC1) was highly positively correlated with all the variables, except for DW, FW and PT. It is interesting to notice that the scatter plot clearly separated the two species into separated groups; however, both ‘Saxa’ and ‘Vertus’ microgreens at LV and 150 PPFD differently separated creating other two sub-groups.

## 4 Discussion

So far, different species and cultivars have been tested as possible candidates for food production in space according to some specific requirements, including their nutritional values, resource-use efficiency and suitability to grow in a closed controlled environment (De Pascale et al., 2021). Therefore, it is fundamental to investigate the interaction among environmental variables on microgreens growth, development, and phytochemical production.

### 4.1 Species-related differences

In the present study, the two species tested, *B. oleracea* ‘Vertus’ and *R. raphanistrum* ‘Saxa’, although both belonging to the Brassicaceae family, showed a very different growth, morpho-anatomical development, and bioactive compounds level. ‘Saxa’, which was harvested first (10 days after sowing vs. 11 for ‘Vertus’), reached the highest fresh and dry weight as well as the highest elongation (**Table 1**, **Figure 2**). This could be explained by the anatomical analysis (**Table 3**), which highlighted a thicker mesophyll in ‘Saxa’ compared to ‘Vertus’, due to a higher palisade and spongy thickness and also due to a higher percentage of intercellular spaces. Leaf anatomical structure has a fundamental relationship with the plant photosynthetic performance, and usually a thicker and more dense tissue facilitates the transfer of gas and water among mesophyll cells (Amitrano et al., 2022). Moreover, palisade cells are also known to concentrate more chloroplasts compared to spongy cells (Nguyen et al., 2019), so one would expect ‘Saxa’ to have a higher photosynthetic pigment content. In our study, however, the content of both chlorophylls and carotenoids, as well as neoxanthin and lutein, was higher in ‘Vertus’ (**Table 5**). This is probably ascribed to the reduced palisade thickness of ‘Vertus’ leaves, which may force the plant to synthesize more pigments in order to intercept more light, thus increasing the photosynthetic efficiency. ‘Vertus’ also presented the highest content of dehydroascorbate, ascorbate and polyphenols compared to ‘Saxa’ (**Table 2**). Species-specific differences can play a significant role in determining the content of ascorbate in different plants (Davey et al., 2000). Ascorbate (or vitamin C) is a well-known antioxidant and radical scavenger and is an essential dietary nutrient in humans. Its role in human body involves the enhancement of the immune system (Crucian et al., 2018), prevention of bone density loss (Weber, 1999) and a function as a cofactor in the biosynthesis of collagen, carnitine, and neurotransmitters (Naidu, 2003). Recently, vitamin C has also been reported to have a role as radioprotector (Cai et al., 2001; Yamamoto et al., 2010; Mortazavi et al., 2015), thus in a space mission context, can help neutralizing free radicals and protecting cells from damage induced by ionizing radiation (Gómez et al., 2021). Another difference between the two species concerned the accumulation of minerals (**Table 6**). ‘Saxa’ was found to be a higher accumulator of nitrates and phosphate, while ‘Vertus’ accumulated more sulfate. It is noteworthy that the nitrate content in both plant species was well below the maximum levels allowed in food products by the European Commission regulation No 1258/2011. Therefore, the accumulation of these compounds for the two microgreen species under the above-reported growing conditions does not represent a health concern (Amitrano et al., 2021c).

### 4.2 Light intensity influence on microgreen development and phytochemical content

Different studies have shown that light intensity has a significant impact on the antioxidant potential of microgreens in terms of anthocyanins and polyphenols content, usually with increments in light corresponding to increments in the phytochemical profile (Samuolienė et al., 2013; Craver et al., 2017). Here we found that the highest light intensity (300 PPFD) was associated with a higher ascorbate content (**Table 4**), consistently with previous research demonstrating a progressive ascorbate accumulation in microgreens, proportionally to the light level (Jones-Baumgardt et al., 2020). However, in our study we did not find a significant variation in the content of anthocyanin or polyphenols in microgreen grown under different light irradiances, nor in the carotenoids and other photosynthetic pigment content. Secondary phytochemicals, such as polyphenols and anthocyanins, are highly desirable in astronaut’s diet since they have several positive impacts on health, including cardiovascular health, neuroprotective function, diabetes management, anti-inflammatory and anti-cancer activity (Gonçalves et al., 2021) and could help counteract the detrimental effect of space environment (Gómez et al., 2021). Carotenoids are also essential for human nutrition; incorporating carotenoids into the food supply for space missions could have multiple benefits, including antioxidant protection (Palozza and Krinsky, 1992), immune system support (Chew and Park, 2004), and eye health maintenance (Ma et al., 2012). The fact that these compounds (anthocyanins, polyphenols and carotenoids), which are also desirable for nutritional purposes, do not increase with light intensity represents an added value as there is always a trade-off between light intensity and electricity consumption in controlled environment agriculture (Kobayashi et al., 2022), especially in the sight of space outposts oriented towards eliminating or minimizing superfluous wastes (Poulet et al., 2014). Moreover, in our study, microgreens at 150 PPFD grew taller than those at 300 PPFD, probably because of a stretching mechanism, typical of plants grown under low light intensity, which lengthen towards the light source in order to enhance their light harvesting capacity (Colonna et al., 2016).

It is worth noting that the lower light intensity did not impact the total fresh weight of microgreens which was still comparable to that produced at 300 PPFD (**Table 1**). The dry weight, however, as well as the dry matter content, increased under the high light intensity. This is a common response of plants grown under high irradiance and is also consistent with the enhanced thickness of the palisade tissue under 300 PPFD (**Table 3**). The increase in palisade tissue thickness goes in parallel with the development of more chloroplasts, thus with increments in the photosynthetic rate (Oguchi et al., 2005). Besides, being the site of photosynthesis, chloroplasts also represent a site for starch storage in the form of grains (Zeiger et al., 2002). Indeed, in our study at 300 PPFD, together with the increments in palisade tissue, the total NSC levels increased (**Table 5**). The two species showed to be accumulators of soluble carbohydrates, since soluble sugars (glucose, fructose, sucrose) dominated the total non-structural carbohydrate content. Starch was always much lower than soluble sugars, but its content varied more in response to the environmental variables tested. In almost all photosynthetic organisms’, starch is one important products of photosynthesis and the storage carbohydrates in leaves, allowing the accumulation of carbon and energy without affecting the osmolyte concentration of the cell. (Malinova et al., 2018). Starch is accumulated in chloroplast during the day and degraded at night to support plant needs, with a complex pathway requiring the coordinated actions of several enzymes (Stettler et al., 2009). Even though we cannot go deeper into the starch kinetics, here, we can hypothesize that, as for other species like *Arabidopsis*, *Lemna*, and mung bean (Yehu et al., 2015; Mengin et al., 2017; Amitrano et al., 2020b), under high irradiance microgreens allocate photosynthates to starch, as a response to an imbalance between photosynthate production and use. This shows that plant metabolism as early as 10-11 days after germination was already fully dominated by photosynthetic production of assimilates, highlighting the importance of the environmental control on gas exchanges as a tool to govern plant performances even in microgreens production.

### 4.3 VPD levels influence on microgreen development and phytochemical content

The air VPD is the primary factor affecting the transpiration rate of crops in controlled environment, thus affecting plants’ photosynthetic rate and the whole development of the leaf lamina (Carins Murphy et al., 2014; Amitrano et al., 2019). It has been demonstrated that plants grown under high VPD levels reduce the photosynthesis and the biomass production and are often characterized by a lower leaf thickness and a reduction of cell enlargement (Aliniaeifard et al., 2014). Our results partially agree with this statement, since HV microgreens did not show significant difference in growth in term of fresh and dry weight, only resulting shorter than LV microgreens (**Table 1**, **Figure 2**). However, HV microgreens showed a reduced tissue thickness and a higher percentage of intercellular spaces, compared to LV (**Table 3**). As mentioned before, having more intercellular spaces can represent a disadvantage for HV microgreens, already subjected to an enhanced transpiration due to the dryer air (high VPD). Indeed, the wide presence of intercellular spaces reduces the cell connection, decreasing the velocity of both gas and water flows, thus imposing a high mesophyll resistance (Amitrano et al., 2019). In accordance with our results, in a previous study on *V. radiata* sprouts subjected to different levels of relative humidity, sprouts at low RH (high VPD) evolved morpho-anatomical traits which increased the resistance to water flows and did not allow them to elude water loss and allocate biomass (Amitrano et al., 2020b). Here, HV condition did not negatively impact microgreen biomass accumulation, but the leaf anatomy developed by HV microgreens characterized by a higher resistance of gas and water flows, would probably lower the photosynthetic and hydraulic efficiency in future plant growth phases.

Furthermore, we observed that HV microgreens had a greater content of ascorbate. To our knowledge there is no previous research on the effect of VPD on microgreens. Our results could be compared with findings from Amitrano et al., (2021c) which reported an increase in total ascorbate content in lettuce grown at 1.76 kPa in comparison to 0.69 kPa, due to the mild stress given by the high VPD conditions. Usually, when plants are subjected to mild stresses caused by high temperature, salinity, VPD or drought, they tend to synthesize more antioxidants with scavenger activity, trying to react to the oxidative stress. The production of antioxidants is effective above a threshold depending on the type and intensity of the stress. Exceeded the threshold, plants are not able to react anymore with their biochemical activities and start to perish (Lihavainen et al., 2016).

Here we also found that the high VPD level reduced the content of nitrates in both species. This effect is useful in the sight of cultivating microgreens for nutritional purposes. Usually, lower content of nitrates is also associated with the qualitative parameters with respect to the yellowish of the leaves, and in particular with enhanced L*, b* and chroma and reduced hue (Navarro et al., 2012). In this study, there were no significant differences between HV and LV microgreens concerning L* and chroma; however, microgreens at HV showed an enhanced yellowness exemplified by the parameter b* and by a reduced chroma. For edible products the analysis of color is an important tool since consumers are easily influenced by the qualitative aspect of vegetables (Jha, 2010). Furthermore, several studies have found an inverse relationship between b* and dry weight, using the b* parameter as a proxy for stress level (Ibraheem et al., 2012; Dodig et al., 2019). LV microgreens with a reduced yellowness in both species achieved a better visual quality, they also maintained high levels of polyphenols, vitamin C and anthocyanins and enhanced the accumulation of pigments such as neoxanthin, lutein and β-carotene, compared to HV (**Table 5**). These pigments, besides having important nutritional functions, are used as proxies for the photosynthetic performance of the plant. Carotenoids are primarily involved in the protective mechanisms of the photosynthetic apparatus from photo-damages (Demmig-Adams and Adams, 2006). Lutein and neoxanthin are involved in the xanthophyll cycle and ease the distribution of absorbed light energy in the leaf, therefore are correlated with the photosynthetic activity (Wong et al., 2020). Thus, LV microgreens not only grew taller, raising the phytochemical content, but also had a more efficient functioning, demonstrated by the more suitable anatomical structure and the high content of pigments and if such a trend is maintained, probably adult plants may have a greater photosynthetic performance. However, the combination of more environmental factors together, can have synergistic or antagonistic effects on microgreen growth and phytochemical content.

### 4.4 Microgreens response to microenvironment and general conclusions

The combination of VPD and light intensity influenced the growth, quality, and the morpho−anatomical traits of the two species. As highlighted by the multivariate analysis performed on all the traits analyzed (**Figure 4**), the genetics had a remarkable effect since ‘Saxa’ and ‘Vertus’, at different VPDs and light intensities, separated into two groups mainly driven by the species. However, both microgreens’ species separated into two different sub-groups due to the effect of LV and 150 PPFD. ‘Saxa’ LV 150 was mainly driven by spongy thickness, violaxanthin and sulfate content, while ‘Vertus’ LV 150 due to the remaining pigments content. Both species grown at LV under 150 PPFD achieved the highest height and fresh weight. Even if they reduced the dry weight compared to microgreens grown under 300 PPFD, they managed to achieve a dry matter content (DM%) comparable to those grown under the highest light intensity (**Figure 1**). DM%, referred to as tissue density, is a good predictor of resource capture and use (Shipley and Vu, 2002). Tomlins et al. (2012) found in sweet potato correlation between the dry matter content and the carotenoids content, attributed to textural changes: plants with high β-carotene were also more solid and less tender, thus preferred by consumers. Our results agree with this research since both ‘Saxa’ and ‘Vertus’ LV 150 increased the tissue thickness and synthetize high β-carotene and photosynthetic pigments (**Table 6**). Moreover ‘Vertus’ LV 150, was the one treatment to develop the highest content of ascorbate, as aforementioned fundamental for nutritional properties in space environment.

In conclusion, it is notable that the effects of environmental factors on the development and the antioxidant potential of microgreens can be complex and dependent on multiple factors. Thus, the modulation of environmental parameters during the early morphogenesis represents a valuable tool to adjust the quality of the products and the plant architecture to fulfill specific needs like the production of more compact plants with high phytochemical content to use in controlled environment systems (Demotes-Mainard et al., 2016; Mah et al., 2018).

For these reasons, to allow microgreen cultivation in space as food supplements, the development of space-targeted agro-technologies is mandatory, taking in mind the possible alterations induced by space flight environments. Indeed, the cultivation in closed facilities of limited volume under microgravity may change microgreen resource utilization, likely modifying the nutritional properties of the products, with species-specific responses. In the light of the above, choosing a low VPD together with a low light intensity might be a good strategy to save energy, while developing microgreens with a high phytochemical content, important for nutritional values and as good anatomical traits indicators of high resource use efficiency, useful also if some of these species could be grown as adult plants to be used as bio-regenerators or for seeds production, and not only as microgreens for dietary supplements. Indeed, it must be taken into account that crop systems like microgreens need a high number of seeds, which can represent a significant upload mass influencing mission planning and payload design, especially in short-duration missions (De Micco et al., 2009). The quantity of seeds to be transported depends on the mission duration, the production capacity of the facilities, the number of crew members, and the average daily consumption of microgreens. Therefore, it is a parameter that needs to be considered specifically for each mission case. Thus, having some plants dedicated to seed production can be an added value; however, in our experiment, the quantity used (6-7 g per trays) is capable of producing a fresh biomass of 1.09 and 1.36 kg and considering 50g per meal it is possible to provide an approximate of 20 meals. Thus, 1 kilogram of seeds is sufficient for a 3-month production representing an efficient volume expenditure.

## Data availability statement

The raw data supporting the conclusions of this article will be made available by the authors, without undue reservation.

## Author contributions

VM, YR, RP, AB, SDP supervision, conceptualization, resources and coordination; SDP, MB funding acquisition, CA and GL run the experiments, the sampling and the biometric and anatomical analysis; GP, SM, SP run the biochemical analysis; CA and GP worked on data curation and on the manuscript preparation; All authors contributed to the article and approved the submitted version.
